# Activation of ER stress mediated by HspB4/Cryaa reduction contributes to photoreceptor apoptosis in *rd9* mouse model of X-linked Retinitis Pigmentosa

**DOI:** 10.1038/s41419-026-09191-1

**Published:** 2026-08-18

**Authors:** Mingzhu Yang, Ruiqi Qiu, Shun Yao, Weiping Wang, Guangming Liu, Jingyang Liu, Bo Lei, Xiuxiu Jin

**Affiliations:** 1 Henan Eye Hospital, Henan Provincial People’s Hospital, People’s Hospital of Zhengzhou University, Zhengzhou, Henan China; 2 Eye Institute, Henan Academy of Innovations in Medical Science, Zhengzhou, Henan China

**Keywords:** Disease genetics, Apoptosis

## Abstract

X-linked Retinitis Pigmentosa (XLRP) is an inherited retinopathy predominantly caused by *RPGR* mutations, ultimately leading to photoreceptor apoptosis and vision loss. Although the genetic basis is well established, the downstream pathogenic and molecular mechanisms remain inadequately elucidated. The aim of this study is to explore the pathogenesis from the perspective of endoplasmic reticulum (ER) stress, which was previously underexplored. Using *rd9* mouse retinas and mouse embryonic fibroblast (MEF) cells, we confirmed the presence of ER stress, activation of the unfolded protein response (UPR), and photoreceptor apoptosis through Western blot and immunofluorescence (IF) analyses. Furthermore, knockdown of Cryaa in 661W cells directly induced ER stress and UPR activation. Transmission electron microscopy (TEM) photomicrographs revealed significant ultrastructural alterations in ER and mitochondria within *rd9* retinas and MEF cells. Proteomic analyses of *rd9* retinas identified significantly enriched pathways including UPR, protein folding, and unfolded protein binding, associated with Cryaa downregulation. Collectively, our results identify Cryaa as a critical regulator of ER stress and demonstrate that its reduction promotes UPR activation and subsequent photoreceptor apoptosis in the *rd9* model. These findings reveal, for the first time, a key role for Cryaa in XLRP pathology and may provide a novel therapeutic perspective for this disease.

## Introduction

Retinitis pigmentosa (RP) is a large heterogeneous grouping of inherited retinal degenerative diseases characterized by photoreceptor cell death and eventual blindness. One of the most severe forms is caused by mutations in the retinitis pigmentosa GTPase regulator (*RPGR*) gene, which account for approximately 75% of XLRP cases [1]. Retinal degeneration 9 (*rd9*) mouse harbors a mutation in the retina-specific *RPGR* Open Reading Frame (ORF) 15 gene, located on the X chromosome, and serves as a valuable model for XLRP [2]. This model is marked by a 32-base-pair duplication in ORF15, introducing a premature stop codon that leads to loss of functional RPGR protein and gradual photoreceptor degeneration [3]. The primary features of *rd9* and deficiency of Rpgr^ORF15^ protein have been comprehensively validated in prior researches [3, 4]. Therefore, the *rd9* mouse serves as a relevant and instructive model for investigating the retinal pathophysiology associated with XLRP.

ER stress is increasingly recognized as a pivotal contributor to the pathogenesis of various retinal degenerative diseases. The ER is essential for maintaining protein homeostasis, being primarily responsible for protein synthesis, protein folding and post-translational modification [5]. Accumulation of unfolded or misfolded proteins triggers UPR, which is mediated by three key sensors: IRE1, PERK, and ATF6 [6]. These pathways function to mitigate ER stress by reducing protein synthesis and enhancing the expression of chaperone proteins. However, prolonged ER stress could result in apoptosis, thereby contributing to the pathophysiology of disorders such as RP and diabetic retinopathy [7–9]. In *rd9* retinas, unfolded or misfolded Rpgr proteins are likely to cause the occurrence of ER stress. Furthermore, emerging evidence suggests that the deficiency of heat shock protein B4 (HspB4) may stimulate ER stress and mitochondrial oxidative stress in cataract [10, 11].

HspB4, also known as αA-Crystallin (Cryaa), is a small heat shock protein traditionally recognized for maintaining lens transparency. Besides, Cryaa functions as a molecular chaperone, preventing the aggregation of denatured proteins and promoting cell survival under stress [12]. In the retina, Cryaa is expressed in photoreceptors and has been shown to mitigate oxidative stress, inhibit apoptosis, and regulate autophagy [13, 14]. Additionally, its deficiency leads to inflammatory responses and activation of apoptotic and necrotic pathways, thereby contributing to photoreceptor degeneration [15]. Although its specific client proteins in photoreceptors are not fully mapped, studies in neurons and retinal pigment epithelium suggest Cryaa interacts with key components of the proteostasis network, including those within ER [16–18]. As a chaperone, Cryaa likely assists in folding or stabilization of proteins critical for photoreceptor function. Its deficiency may lead to the accumulation of misfolded proteins, thereby promoting ER stress.

Photoreceptor apoptosis represents a common pathological endpoint of RP induced by pathogenic gene mutation. Despite significant progress in elucidating the mechanisms of photoreceptor apoptosis, including structural disruptions of the outer segment, visual cycle disturbances, rhodopsin transport disorders, aberrant phototransduction, and altered cGMP levels [19–21], the specific pathogenic and molecular mechanisms of XLRP caused by *RPGR* mutations remain insufficiently understood. Herein, we identified the critical roles of ER stress and UPR in driving photoreceptor apoptosis in *rd9* retinas. Moreover, we verified that the knockdown of Cryaa is sufficient to induce ER stress and UPR. Proteomic analyses of *rd9* retinas corroborated these findings, revealing significant enrichment of pathways related to the UPR, protein folding, and unfolded protein binding associated with Cryaa reduction. Through a combination of molecular biology experiments and proteomic profiling, this research establishes that Cryaa deficiency promotes ER stress and photoreceptor apoptosis in *rd9* mouse model. These findings contribute to unravel pathogenic mechanisms of photoreceptor apoptosis, thus potentially providing new hints for prospective XLRP therapeutic strategies.

## Materials and methods

### Animal care and use

*Rd9* (C57BL/6J-Rpgr^*Rd9*^/BocJ) mice were purchased from Jackson Laboratories (Strain#: 003391, Bar Harbor, ME, USA). The validation of the *rd9* mouse model has been comprehensively documented in previous research [3]. C57BL/6 mice, serving as the wild type (WT), were procured from Vital River Laboratory Animal Technologies Co., Ltd. The animals were maintained under a 12-h light-dark cycle and were provided with a standard chow diet. The care of animals adhered to the guidelines established by the Association for Research in Vision and Ophthalmology (ARVO). All experimental procedures involving animals received approval from the Ethics Committee of Henan Eye Hospital (Permit No: HNEECA-2022-16). No formal statistical power calculation was performed prior to the study. The sample size for each experimental group was chosen based on established standards in the field for similar phenotypic and biochemical analyses [22], which has been demonstrated to be sufficient to detect significant and biologically relevant differences. Pre-established criteria were applied for the inclusion of animals. Only animals with a confirmed genotype and completing the entire experimental protocol without severe health issues unrelated to the model were included in the analysis. No randomization was used to allocate animals to experimental groups. Allocation was based entirely on genotype and ages *(rd9* or WT, 2 m or 9 m). All animals of the same genotype and age from the same litter were assigned to the same experimental group. Blinding was not implemented in this study due to the operational requirements of genotype-specific procedures and animal care. To ensure objectivity, all results were analyzed using GraphPad Prism, which minimized subjective interpretation.

### Construction of primary cultured MEF cells

WT or *rd9* embryos were harvested at embryonic day 14 and immediately placed in ice-cold phosphate-buffered saline (PBS). Following the removal of the liver, spleen, and intestine, the embryonic bodies were finely minced and subsequently subjected to digestion using 0.25% Trypsin-EDTA (25200056; Gibco, NY, USA). The incubation with trypsin-EDTA was terminated by the addition of complete MEF medium, consisting of high-glucose DMEM supplemented with 10% fetal bovine serum (FBS) and 1% penicillin/streptomycin. The digested tissue solutions were filtered using a sterile 40 μm cell strainer (352340, BD Falcon, Durham, USA) and washed with cold PBS. MEF cells were collected by centrifugation at 1000 rpm for 5 min and then cultured at 37 °C in a 5% CO_2_ incubator. The culture medium was refreshed every two days, and MEF cells were passaged upon reaching 90% confluency. For the purposes of this study, MEF cells at passage 3 were utilized.

### Cryaa small interfering RNA (siRNA) transfection and plasmid overexpression in 661W cells

The 661W cells used in this study were obtained from BNCC (BeNa Culture Collection, BNCC353554, Xinyang, China). All cells used in this study were authenticated by short tandem repeat (STR) profiling and routinely tested to confirm the absence of mycoplasma contamination prior to experimentation. 661W cells were cultured in high-glucose DMEM (Corning, 10-013-CVRC, China) containing 10% FBS (Corning, 35-081-CV, New Zealand). To knockdown Cryaa expression in 661W cells, a pool comprising 3 distinct siRNA duplexes targeting mouse αA-crystallin (sc-40431, Santa Cruz, Dallas, USA) was utilized. A negative control (NC) siRNA, provided by the manufacturer (GentleGen, Suzhou, China), was employed. The specific sequences of the siRNAs are detailed in Supplementary Table S1. For the transfection of 661W cells, siRNA (targeting Cryaa or a non-targeting control) was mixed with the HiPerFect transfection reagent (301705, Qiagen, Valencia, USA) in accordance with the manufacturer’s protocol. The 661W cells were then incubated with the transfection complexes under standard growth conditions. Total protein extraction was performed 70 h post-transfection. The pcDNA3.1(+)/Cryaa was constructed and applied to transfect 661W cells and MEF cells for Cryaa overexpression in subsequent experiments, with an empty plasmid serving as a negative control.

### Transmission electron microscope (TEM)

The eyeballs of WT and *rd9* mice were enucleated and fixed in electron microscopy fixative solution (BP0130, Biossci, Wuhan, China) for 2 h. Photoreceptor samples, measuring 1 mm × 1 mm × 1 mm, were isolated from the retinal sections. These samples were then washed with PBS and fixed in a 1% osmium tetroxide solution prepared in a 0.1 M phosphate buffer (pH 7.4) at ambient temperature for 2 h. The samples underwent dehydration through a graded series of ethyl alcohol and were embedded in 812 epoxy resin (SPI, 02660-AB, PA, USA) overnight at 37 °C. The embedded tissues were polymerized over a period of 48 hours at 60°C. Ultra-thin sections, approximately 80 nm, were sectioned using a Leica UC7 ultramicrotome and stained with a 2% uranyl acetate saturated alcohol solution for 10 min, followed by staining with 2.6% lead citrate for an additional 10 min. Pathological alterations in ER and mitochondria were examined using a TEM (HT7800, Hitachi, Tokyo, Japan). MEF cells derived from WT or *rd9* mice were digested, collected, and preserved in the electron microscopy fixative solution. All cells were processed for serial sectioning and mounted on copper mesh grids for TEM analysis.

### Western blot analysis

Retinas were excised from the eyeballs of WT and *rd9* mice and homogenized in RIPA lysis buffer (EpiZyme, Shanghai, China) supplemented with a 1% protease inhibitor cocktail (EpiZyme). The protein concentration was determined using a BCA protein assay kit (Beyotime). Proteins were then subjected to electrophoresis on a 10% SDS-polyacrylamide gel and transferred onto polyvinylidene difluoride (PVDF) membranes (Millipore, Burlington, MA, USA). The PVDF membranes were blocked with 5% non-fat milk for 1.5 hours, followed by an overnight incubation with antibodies specific to Cryaa (ab181866, Abcam), XBP1s (24868-1-AP, Proteintech), Atf4 (ET1612-37, Huabio), Atf6 (ab227830, abcam), CHOP (ET1703-05, Huabio), Bcl-2 (ET1702-53, Huabio), Bax (ET1603-34, Huabio), GRP78 (HA722202, Huabio), p-EIF2S1 (ET1603-14, Huabio), EIF2S1 (HA722112, Huabio), β-tubulin (EM0103, Huabio) and β-actin (ab8227, Abcam) at 4 °C. β-tubulin or β-actin served as the endogenous reference. Each group contained three replicates. Full and uncropped Western blots were uploaded as Supplemental Material (see Original Western Blots).

### Electroretinography (ERG) assessment

Retinal function was assessed using ERG as previously described [22]. Mice underwent overnight dark adaptation before being anesthetized via intraperitoneal injection of 1.25% avertin (0.4 ml/20 g body weight) (Aibei, Nanjing, China). Tropicamide eye drops (Santen, Osaka, Japan) were administered to induce pupil dilation 5 min before recording. All procedures were conducted under dim red light to facilitate the acquisition of full-field ERGs using the RetiMINER-C visual electrophysiology system (AiErXi, Chongqing, China). For dark-adapted ERGs, stimulus intensities ranged from −3 to 1 log cd-s/m², while light-adapted ERGs were recorded with stimuli ranging from 0 to 1 log cd-s/m² following a 5-min light adaptation period. The amplitudes of the a- and b-waves were analyzed to evaluate retinal function.

### Optical coherence tomography (OCT) examination

The assessment of retinal function was conducted using OCT, following a method previously described in the literature [23]. Age-matched *rd9* and WT mice were anesthetized with a 1.25% avertin solution at a dosage of 0.4 mL per 20 g of body weight. Pupillary dilation was achieved by administering compound tropicamide eye drops five minutes prior to measurement. Retinal structural images were captured using a Micron IV^®^ OCT system (Phoenix Research Labs, Pleasanton, CA, USA). The specific OCT setup parameters included: Position (778), Paddle position (371), Contrast (43), Gamma (26), Brightness (-17), Coefficient A (50), and Coefficient B (120). Image analysis and measurements were performed using InSight XL v.2 software (Phoenix Research Labs).

### HE staining, TUNEL and immunofluorescent staining

WT and *rd9* mice were euthanized at 2 months or 9 months of age. Hematoxylin and eosin (HE) staining was performed as outlined before [24]. The eyes were enucleated and fixed overnight using FAS eyeball fixative solution (Servicebio, Wuhan, China). Subsequently, the eyeballs were embedded in paraffin, and serial sections of 3 μm thickness were prepared along the cornea-optic nerve axis. The sections were subjected to staining using a TUNEL assay kit (G1502, Servicebio) or incubated overnight at 4 °C with primary antibodies against α-crystallin A (anti-Cryaa, ab181866, Abcam), Bax (ET1603-34, Huabio), Bcl-2 (ET1702-53, Huabio), XBP1s (24868-1-AP, Proteintech), Atf4 (ET1612-37, Huabio), and CHOP (ET1703-05, Huabio). Following this, the slides were incubated in the dark with DAPI and secondary antibody. Fluorescence was visualized using a fluorescence microscope (Nikon, Tokyo, Japan). The intensity of TUNEL staining was quantified using Image-Pro Plus 6.0 software, and the TUNEL-positive rate was calculated as the ratio of TUNEL-positive cells to DAPI-positive cells, expressed as a percentage.

### Bioinformatic and statistical analysis

Retinas were obtained from *rd9* and WT mice at 2 months and 9 months. Three retinas from each group were collected and processed as previously [24]. Briefly, retinas were lysed and prepared into peptides for liquid chromatography-tandem mass spectrometry (LC-MS/MS). Data from data-dependent acquisition (DDA) and data-independent acquisition (DIA) were processed using ProteinPilot software (version 5.0.1) and SWATH 2.0 plug-in for PeakView software (Version 2.2). Protein intensities were normalized and log2-transformed for further analysis. Statistical significance of protein expression was determined using t-tests to calculate *p* values. Differentially expressed proteins (DEPs) were identified based on a threshold of *p* < 0.05 and a fold change (FC) greater than 1.5. Bioinformatic analysis was conducted as previously outlined [22]. Gene Set Enrichment Analysis (GSEA) analysis was performed using OmicShare tools (https://www.omicshare.com/tools). The sort order of all proteins was set up as Signal2Noise method. The average optical density of immunofluorescent images was measured and calculated by Aipathwell software (Servicebio). Experimental data were statistically analyzed with GraphPad Prism 8 software (GraphPad, San Diego, CA, USA) and expressed as mean ± standard error of mean (SEM). Unpaired *t*-test, one-way ANOVA and two-way ANOVA followed by Bonferroni correction were applied. Statistical significance was defined as *p* < 0.05.

## Results

### The ONL thickness reduction and retinal function decline were observed in *rd9* mice through morphological and functional evaluation

**Fig. 1:**
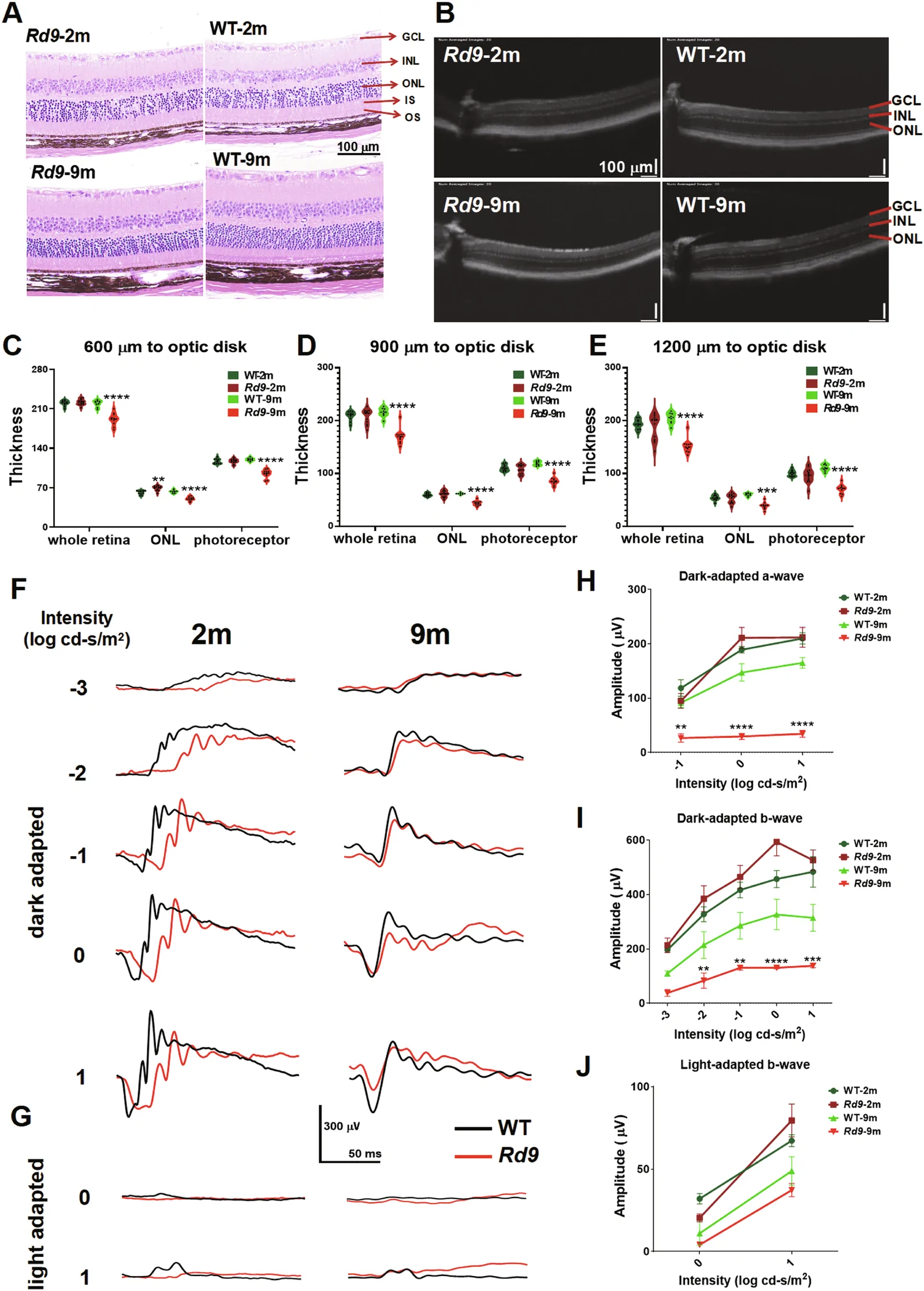
The phenotype of *rd9* mice containing *Rpgr* mutation. **A** Representative retinal images of HE staining in *rd9* and WT mouse retinas at 2 months and 9 months. **B** The OCT images of age-matched WT and *rd9* mice. **C**–**E** The thickness of the whole retina, ONL and photoreceptor in age-matched WT and *rd9* mice at 600 μm, 900 μm and 1200 μm to optic disk. (*n* ≥ 8). **F**, **G** Retinal function of age-matched WT and *rd9* mice were assessed by dark- and light-adapted ERG. **H**–**J** Summaries were provided for the ERG amplitude versus flash intensity profiles of the dark-adapted a-wave, dark-adapted b-wave, and light-adapted b-wave. The results were mean ± SEM. Significance of difference (**p < 0.01, ***p < 0.001, ****p < 0.0001) was determined by using two-way ANOVA with Bonferroni correction. (*n* = 3) ERG electroretinogram, WT wild type, 2 m 2 months, 9 m 9 months, GCL ganglion cell layer, INL inner nuclear layer, ONL outer nuclear layer.

To determine the retinal morphology changes prior to and after disease onset in *rd9* mice, we performed HE staining at 2 m and 9 m. In Fig. 1A, amounts of photoreceptors disappeared in *rd9* retina at 9 m, and the thickness of whole retina and outer nuclear layer (ONL) decreased. Besides, OCT appearances of WT and *rd9* mice retinas were observed at 2 m and 9 m (Fig. 1B). We analyzed and summarized the thickness of whole retina, ONL and photoreceptor in age-matched WT and *rd9* mice at 600 μm, 900 μm, 1200 μm to the optic disk (Fig. 1C, D, E). The thickness of whole retinas in 9 m *rd9* mice revealed significantly decline, especially at distal peripheral retina. The thickness of ONL at 9 m *rd9* exhibited a significant decrease at distances of 600 μm, 900 μm, and 1200 μm from the optic disk. In 9 m *rd9*, there was a marked decrease in photoreceptors thickness (ONL+IS+OS) at 600 μm, 900 μm, 1200 μm. Furthermore, flash ERG was performed to evaluate the retinal functions of age-matched WT and *rd9* mice at 2 m and 9 m. The a-wave is primarily generated by photoreceptors, while the b-wave mainly reflects the activity of bipolar cells [25, 26]. In Fig. 1F, the scotopic ERG responses occurred a delay in the onset of the a-wave and b-wave in *rd9* mice at 2 m and showed a remarkable decrease at 9 m. The dark-adapted amplitudes of a-wave and b-wave were significantly lower in 9 m *rd9* (Fig. 1H, I). The results indicated the photoreceptors function was disrupted in 9 m *rd9*, which corresponds to the morphological changes in *rd9*. As for the photopic ERG, the average amplitudes of b-wave in 9 m *rd9* mice declined without significance (Fig. 1G, J). Overall, the retinal structure and morphology are abnormal, and the retinal functions were incompetent in 9 m *rd9* mice. The above results indicated that the *Rpgr* mutation impaired visual functions obviously at 9 m. These findings are consistent with the previous studies [3, 4].

### Photoreceptor apoptosis was detected in *rd9* mice with retinal immunofluorescent staining

**Fig. 2:**
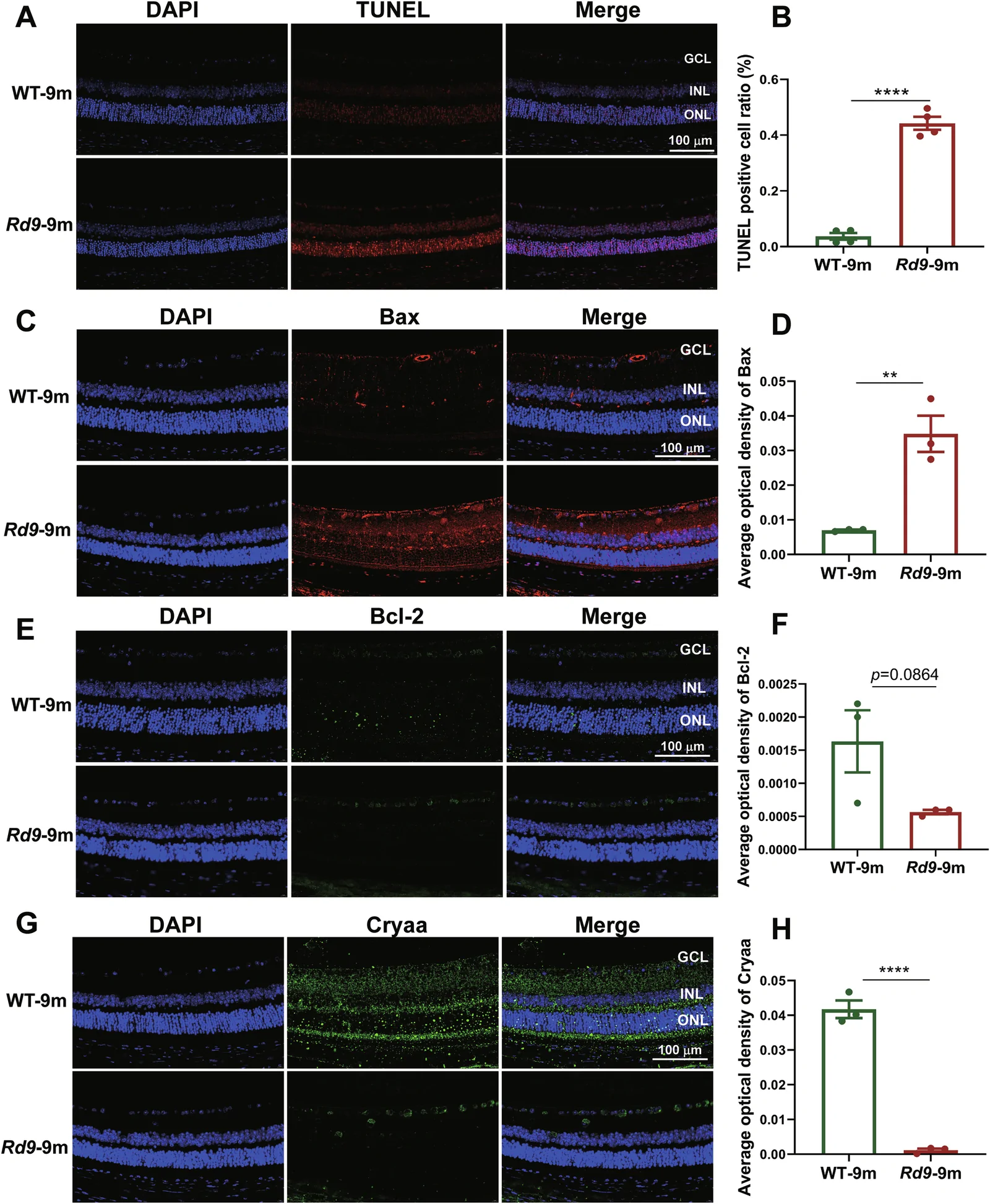
Retinal paraffin sections with immunofluorescent staining in age-matched WT and *rd9* mice. **A** Immunofluorescent staining for TUNEL in WT and *rd9* mice at 9 months (blue: DAPI, red: TUNEL). **B** The corresponding quantification of the TUNEL-positive cell ratio. (*n* = 4, data presented as mean ± SEM). **C**, **D** Representative images of Bax (red) immunofluorescence and average optical density of Bax in 9 m WT and *rd9* mice. **E**, **F** IF staining detected the expression of Bcl-2 (green) in WT and *rd9* mice at 9 m. **G**, **H** Immunofluorescence results of Cryaa (green) at 9 m. (*n* = 3, data presented as mean ± SEM), Scale bar: 100 μm. Significance of difference (**p < 0.01, ****p < 0.0001) was determined by using an unpaired *t*-test.

TUNEL staining was conducted on retinal paraffin sections obtained from WT and *rd9* mice at 9 months of age to evaluate retinal apoptosis (Fig. 2A). Statistical analysis revealed a significantly higher number of TUNEL-positive photoreceptors in the retinas of *rd9* mice (Fig. 2B). Furthermore, the retinal paraffin sections were also detected with immunofluorescence using a Bax antibody. The retinas of *rd9* mice at 9 months showed a significantly higher presence of Bax protein (Fig. 2C, D). Immunofluorescence staining identified Bcl-2 expression in both WT and *rd9* mice. According to Fig. 2E, F, *rd9* mouse retinas had a lower amount of Bcl-2 proteins at 9 months. Immunofluorescence analysis was conducted to assess the expression levels of Cryaa. As illustrated in Fig. 2G, H, a significant reduction of Cryaa levels was observed in *rd9* retinas at 9 months. The experimental results presented above indicated the occurrence of photoreceptor apoptosis in the retinas of *rd9* mice.

### ER stress and UPR pathway proteins were confirmed in *rd9* retinas through Western Blots and immunofluorescence

**Fig. 3:**
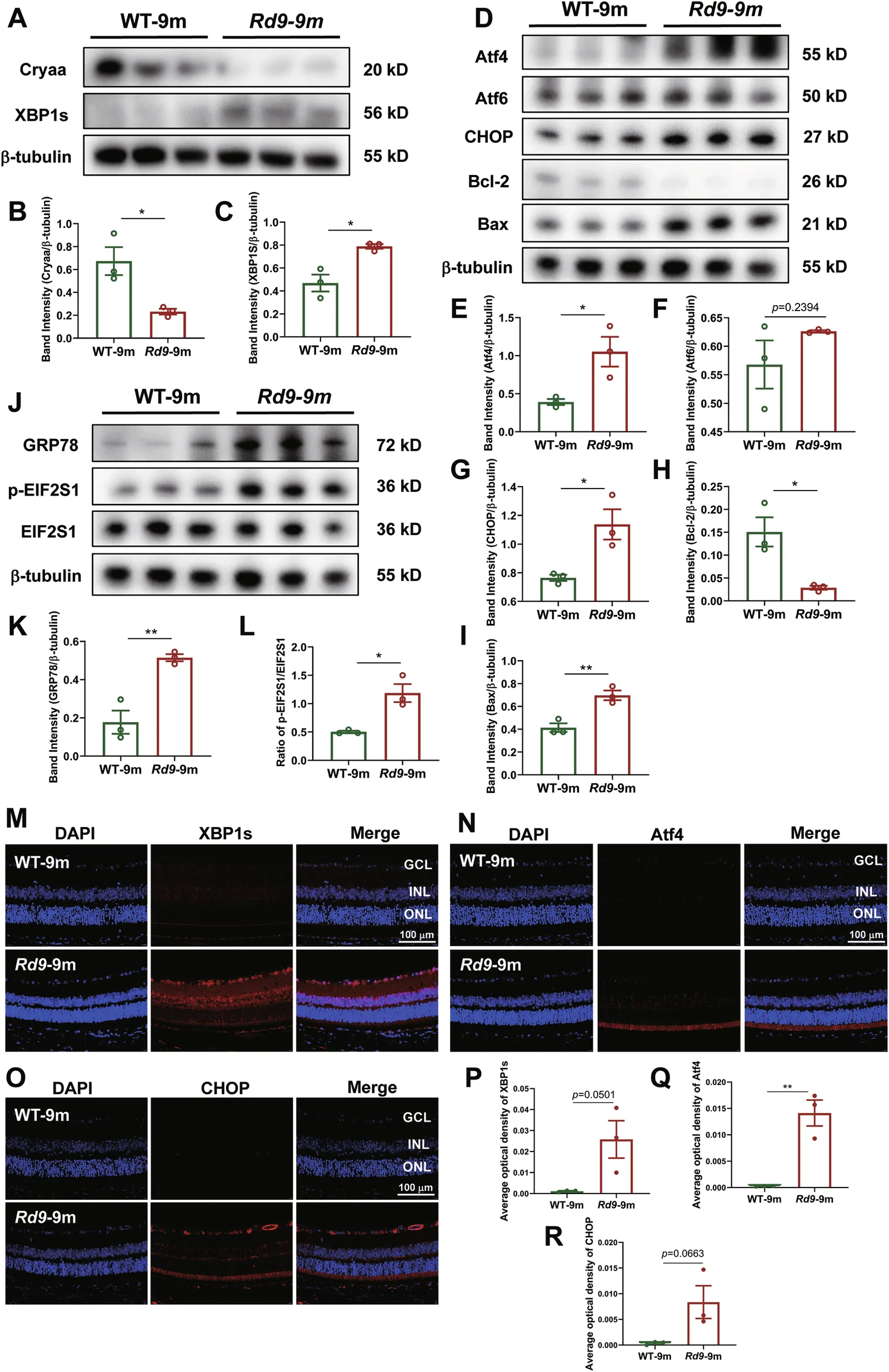
Protein expression levels and immunofluorescence involved in ER stress and UPR pathways in WT and *rd9* mouse retinas. **A**–**C** Western blot validation of Cryaa and XBP1s in WT and *rd9* mice. **D**–**I** Protein levels of Atf4, Atf6, CHOP, Bcl-2 and Bax in age-matched WT and *rd9* retinas. **J**–**L** GRP78, p-EIF2S1 and EIF2S1 expression in WT and *rd9* retinas at 9 months. **M**, **P** Retinal tissue sections with IF staining determined the XBP1s expression and distribution in 9 m WT and *rd9*. **N**, **Q** Immunofluorescent staining results of Atf4 in WT and *rd9* mouse retinas. **O**, **R** Representative immunofluorescent staining of CHOP and average optical density in *rd9* mouse retinas compared to WT. (*n* = 3) The results were mean ± SEM. Significance of difference (*p < 0.05, **p < 0.01) was determined by using an unpaired *t*-test.

To demonstrate whether the ER stress and UPR pathways were activated in *rd9* retinas, we performed Western blotting and immunofluorescence validation using canonical proteins involved in ER stress and UPR pathways, and analyzed their relative gray values. Figures 3A, B, C illustrated the protein levels of Cryaa and XBP1s in 9-month-old retinas. The protein levels of Cryaa in 9-month-old *rd9* retinas were significantly reduced. In contrast, the protein level of XBP1s was elevated in 9-month-old *rd9* retinas. As shown in Fig. 3D, the expression levels of the proteins Atf4, CHOP, and Bax exhibited a statistically significant increase, whereas Atf6 demonstrated an increase with no significance. Conversely, the expression of Bcl-2 showed a marked decrease, as depicted in Fig. 3D, E, F, G, H, I. Apparently, the phosphorylation levels of EIF2S1 and GRP78 were notably up-regulated in *rd9* retinas at 9 months, suggesting activation of the UPR pathway (Fig. 3J, K, L). The immunofluorescence analysis demonstrated an increased expression of XBP1s in *rd9* retinas at 9 months, as illustrated in Fig. 3M, P. Furthermore, Fig. 3N, Q revealed a significantly elevated level of Atf4 in the *rd9* mouse retinas at the same age. As shown in Fig. 3O, R, there was an elevation in CHOP levels in the retinas of *rd9* mice at 9 months of age. The immunofluorescence findings, in agreement with the Western blot analyses, provided additional confirmation of the activation of ER stress and UPR pathway in *rd9* retinas.

### The ER and mitochondria morphological changes observed by TEM in *rd9* photoreceptors

**Fig. 4:**
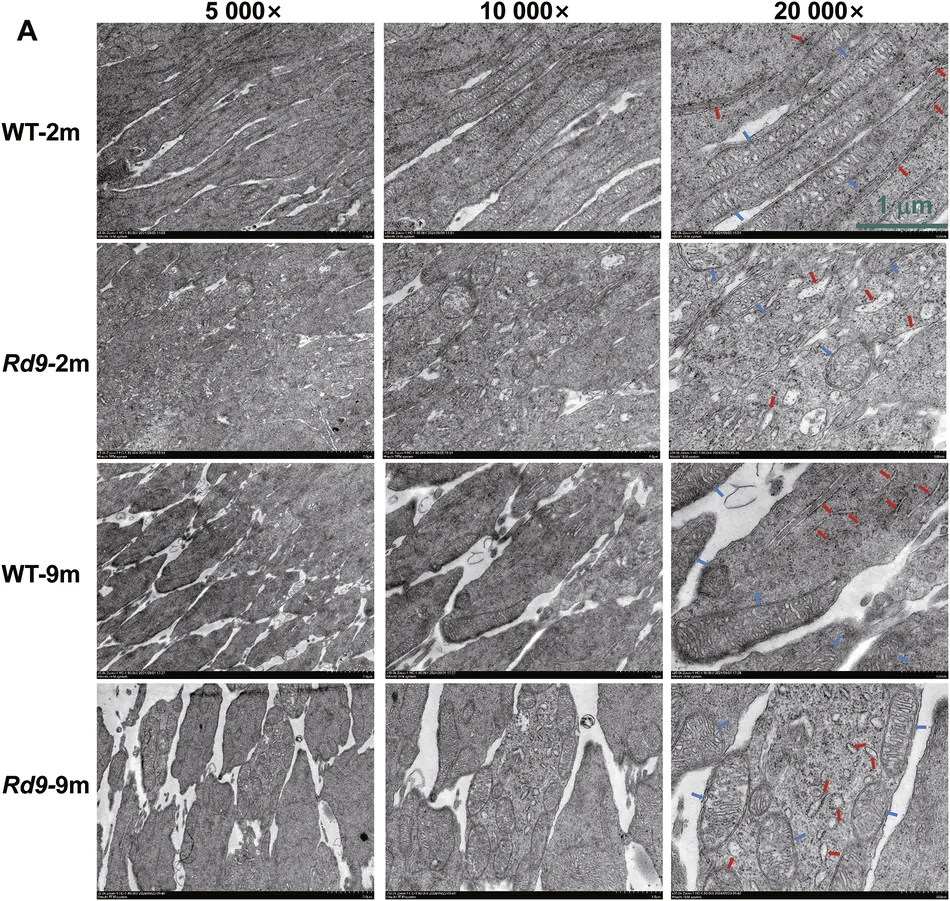
Transmission electron microscopy (TEM) images of mitochondria and ER morphology in retinas of age-matched WT and *rd9*. **A** Representative photomicrographs of photoreceptors at 2m and 9m. *n* =3, red arrows: endoplasmic reticulum, blue arrows: mitochondria, magnification: 5000×, 10000×, 20000×; Scale bar: 1 μm, ER endoplasmic reticulum, 2 m 2 months, 9 m 9 months.

To investigate the effects of ER stress at the organelle level, TEM was used to observe the morphological alterations of retinal ER and mitochondria. ER is an organelle found in eukaryotic cells responsible for the synthesis of proteins, lipids, and oligosaccharides [27]. Upon activation of UPR, ER volume and activity increased. Strikingly, TEM results revealed a significant alteration in the appearance of the ER membrane. The ER exhibited expansion, swelling, and vacuolization, accompanied by a reduction in ribosomal granules, in *rd9* retinas at both 2 months and 9 months. However, rough ER was abundant in the WT group, and the morphology of ER showed intact and normal (Fig. 4A). In addition to pronounced structural alterations in the endoplasmic reticulum, we also observed changes in the mitochondria. The mitochondrial morphology in *rd9* mouse retinas exhibited swelling and abnormalities, characterized by irregular and reduced cristae. The aforementioned results corroborated that both the ER and mitochondria of *rd9* exhibited significant morphological alterations, potentially attributable to ER stress. This further substantiates the presence of ER stress in the retinas of *rd9* mice.

### The knockdown of Cryaa via siRNA in 661W cells activated ER stress and the UPR pathway, and Cryaa overexpression mitigated them

**Fig. 5:**
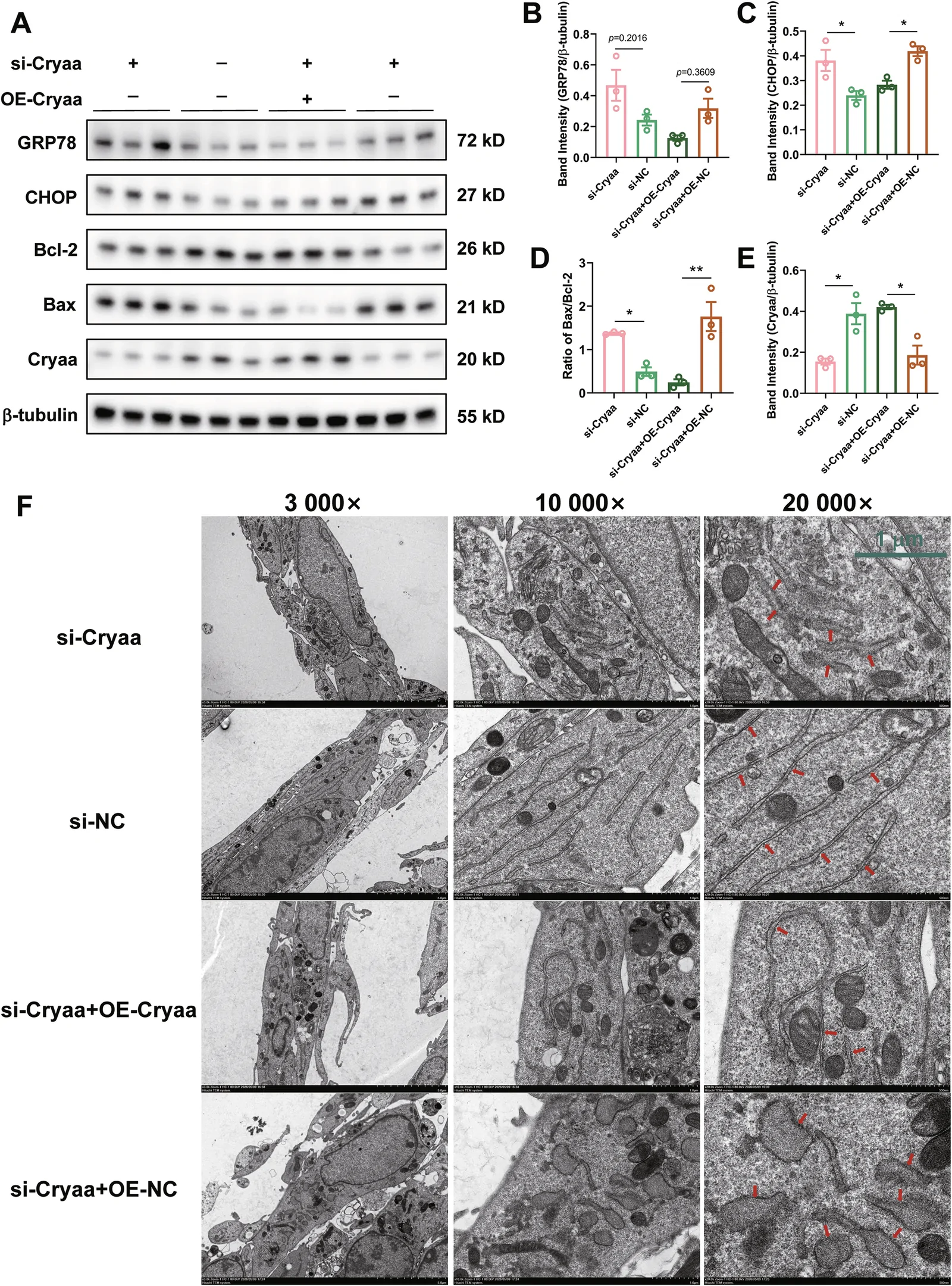
The 661W cells were transfected with Cryaa siRNA followed by Cryaa overexpression (OE), and proteins involved in ER stress were examined by WB, and ER morphology by TEM. **A**–**C** siRNA-mediated knockdown of Cryaa resulted in the up-regulation of GRP78 and CHOP in 661W cells, and Cryaa overexpression reversed them. **A**, **D** WB validated the ratio of Bax to Bcl-2 in 661W cells transfected with Cryaa siRNA and the cells with Cryaa overexpression. **A**, **E** Successful knockdown with Cryaa siRNA and overexpression with Cryaa plasmid were confirmed by WB in 661W cells. Quantification of Cryaa band intensity was conducted. **F** Representative photomicrographs of ER morphology in 661W cells with siRNA or overexpression. red arrows: endoplasmic reticulum, magnification: 3000×, 10000×, 20000×; Scale bar: 1 μm, *n* = 3. The results were shown as mean ± SEM. Significance of difference (*p < 0.05, **p < 0.01) was determined by using one-way ANOVA with Bonferroni correction. si-NC negative control siRNA, OE-NC overexpression with negative control plasmid.

To investigate the role of Cryaa in ER stress and UPR, knockdown experiments and Western blot analyses were performed to assess the expression levels of proteins associated with the ER stress and UPR pathways in 661W cells, a cell line exhibiting photoreceptor characteristics. Figure 5A, D, E illustrated the ratio of Bax/Bcl-2 and the protein level of Cryaa in 661W cells. Following knockdown with Cryaa-siRNA, a significant reduction in the protein level of Cryaa was observed in 661W cells compared to the levels in the negative control (NC) group. Following the transfection with pcDNA3.1(+)/Cryaa, there was a significant elevation of Cryaa protein level. The Western blot analysis indicated an increased expression of Bax protein in the Cryaa-siRNA group compared to the NC group, whereas the expression of Bcl-2 protein was reduced in the Cryaa-siRNA group. Cryaa overexpression partially mitigated these effects. The expression level of CHOP was significantly elevated in the Cryaa-siRNA group compared to the NC, as demonstrated in Fig. 5A, C. Additionally, the expression of GRP78 was higher with no significance in the Cryaa-siRNA group following the knockdown of Cryaa, and showed a decrease in the si-Cryaa+OE-Cryaa group (Fig. 5A, B). Furthermore, Fig. 5F illustrated the alterations of ER morphology in 661W cells following transfection with Cryaa siRNA or overexpression of Cryaa.

### The ER stress validation and ER morphological alterations in primary cultured MEF cells obtained from *rd9* mice

**Fig. 6:**
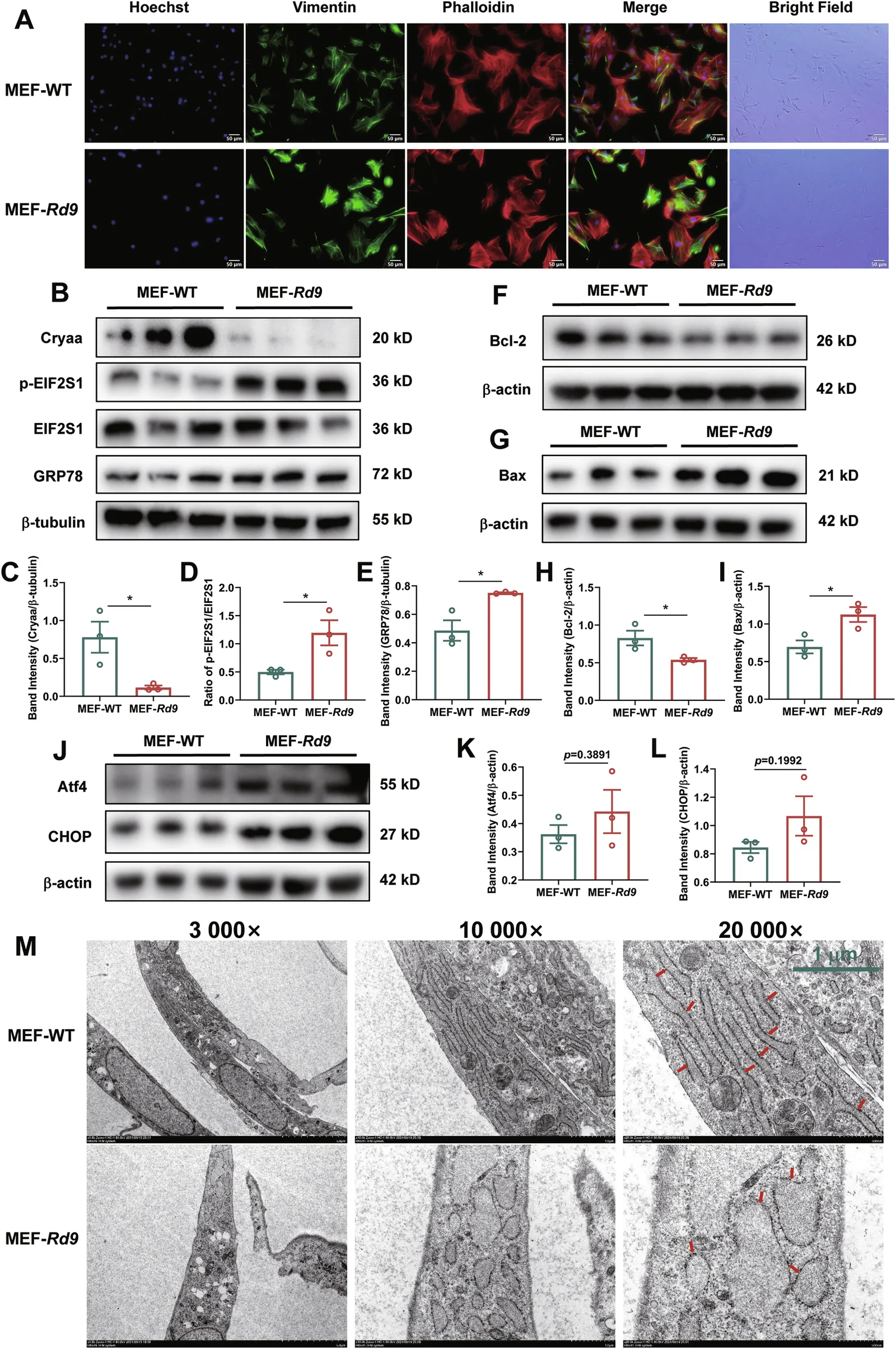
Validation of ER stress in primary cultured MEF cells derived from *rd9* mouse. **A** Identification of MEF cells from WT and *rd9* mice by using immunofluorescence. Hoechst (blue), Phalloidin (red) and Vimentin (green), an antibody specific for MEF cells, were used to validate the purity of MEF cell lines. Bar represents 50 μm. **B**, **C** WB results of Cryaa in MEF-WT and MEF-*rd9* cells. **B**, **D**, **E** Protein levels of p-EIF2S1, EIF2S1 and GRP78 in MEF cells were detected by WB. **F**–**I** WB results showed the reduction of Bcl-2 and up-regulation of Bax in MEF-*rd9* cells compared to MEF-WT. **J**–**L** WB analysis of Atf4 and CHOP expression in MEF-WT and MEF-*rd9* cells. **M** High-resolution transmission electron microscopy images of ER morphology in MEF-WT and MEF-*rd9* cells. 3 duplicates were conducted for each group by WB. The results were mean ± SEM. Significance of difference (*p < 0.05) was determined by using an unpaired t-test. red arrows: endoplasmic reticulum, magnification: 3000×, 10000×, 20000×; Scale bar: 1 μm, ER endoplasmic reticulum.

To further verify the presence of ER stress activation and the downregulation of Cryaa in vitro, we established a mouse embryonic fibroblast (MEF) cell line derived from *rd9* mice and examined the alterations in proteins associated with the ER stress pathway within the MEF cell line. The purity of the MEF cell lines was assessed through immunofluorescence analysis utilizing Vimentin, an antibody specific to fibroblast cells (refer to Fig. 6A). The cytoskeletal structure of the MEF cells was visualized using Phalloidin staining. Furthermore, Western blot analyses were performed to verify the presence of proteins related to ER stress in MEF cells derived from *rd9* and WT mice. Figures 6B, C showed the protein levels of Cryaa in MEF cells, which decreased obviously in the MEF-*rd9* group. The phosphorylation levels of EIF2S1 and the protein levels of GRP78 were up-regulated in MEF-*rd9* cells, as shown in Fig. 6B, D, E. Additionally, when compared to MEF-WT, the Bcl-2 protein levels in MEF-*rd9* cells were significantly reduced, as shown in Fig. 6F, H. Conversely, a significant increase in Bax protein levels was observed in MEF-*rd9* cells, as depicted in Fig. 6G, I. Furthermore, the protein expression levels of Atf4 and CHOP were markedly elevated in MEF-*rd9* group, as demonstrated in Fig. 6J, K, L. The above results indicated that the activation of ER stress pathway existed in MEF-*rd9* cells. Furthermore, TEM analysis revealed that MEF-*rd9* cells displayed a markedly swollen endoplasmic reticulum, in contrast to MEF-WT cells, in which ER exhibited normal appearance (Fig. 6M). These findings indicated that ER swelling and abnormal ER morphology may result from ER stress, and in turn affect ER homeostasis and potentially lead to cellular apoptosis. Moreover, we constructed the pcDNA3.1(+)/Cryaa plasmid and transfected it into MEF-*rd9* cells with MEF-WT as control. Western blot analysis revealed that Cryaa overexpression in these cells reduced the ratio of p-EIF2S1/EIF2S1 and Bax/Bcl-2, and downregulated GRP78 and CHOP expressions, suggesting that Cryaa supplementation could partially alleviate ER stress (Figure. S1).

### Proteomics changes involved in endoplasmic reticulum stress in *rd9* mice

**Fig. 7:**
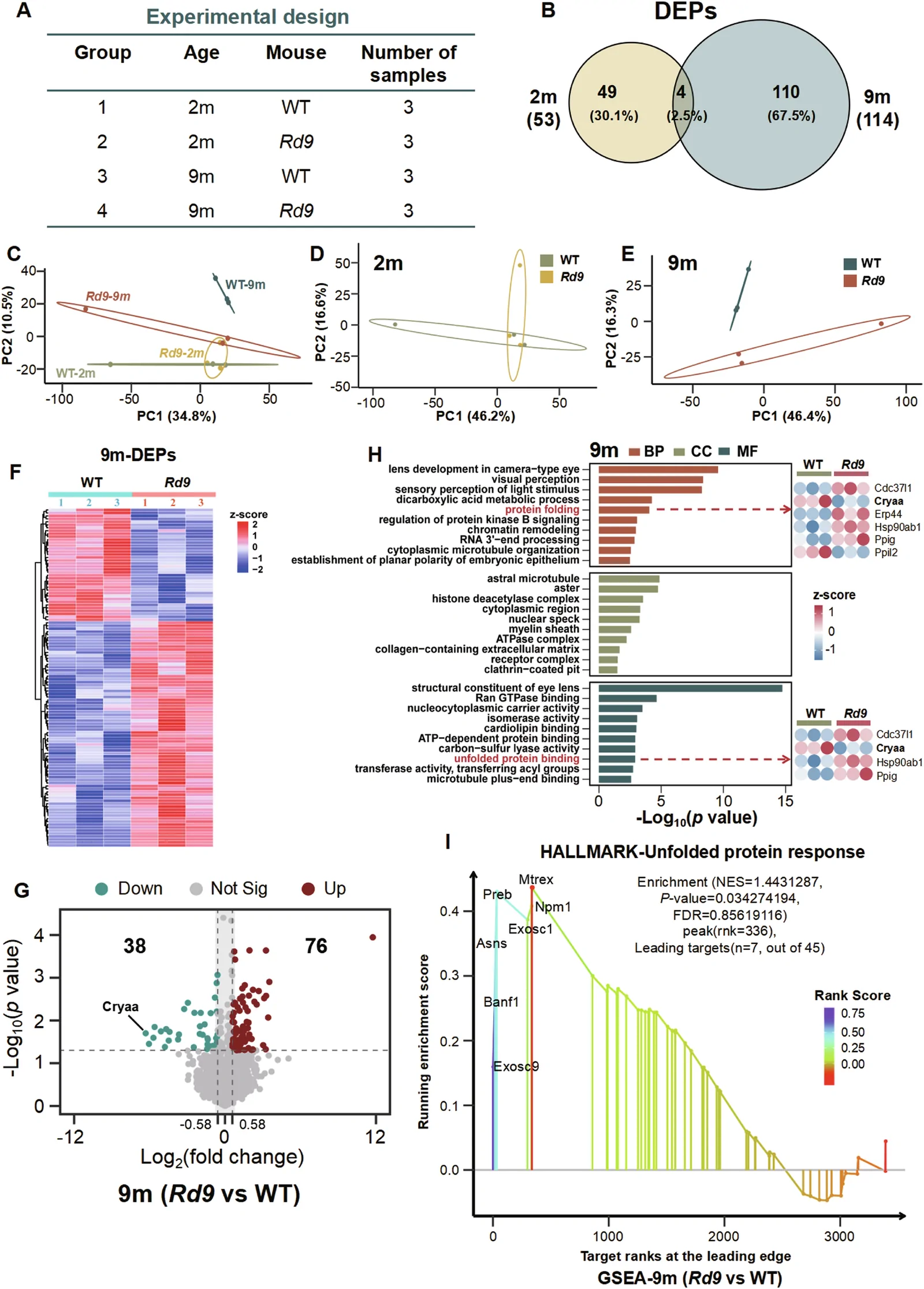
Proteomic analysis of WT and *rd9* mouse retinas. **A** The experimental design. Four groups of retinas were divided into two experiments based on age (2 m or 9 m) and genotype (WT or *rd9*) (*n* = 3 retinas). **B** Venn diagram showed the overlap of DEPs with *p* < 0.05 and FC > 1.5 in retinas at 2 m (yellow) and 9 m (green). **C**–**E** PCA analysis of gross protein differences between samples of WT and *rd9* at 2 m and 9 m. **F** Heatmaps showing DEPs with *p* < 0.05 and FC > 1.5 in 9 m WT and *rd9* retinas. **G** Volcano plot showing 76 up-regulated (red) and 38 downregulated (green) proteins in *rd9* retinas at 9 m (FC > 1.5, and *p* < 0.05). **H** GO enrichment analysis of DEPs at 9 m, including BP, CC, and MF. Heatmaps showing proteins associated with protein folding and unfolded protein binding pathways enriched in 9 m. **I** GSEA analysis of DEPs at 9 m, and the most significantly enriched pathway in the Hallmark database was the unfolded protein response pathway. FC fold change, DEP differentially expressed protein, PCA principal component analysis, GO gene ontology, BP biological process, CC cellular component, MF molecular function, GSEA gene set enrichment analysis, 2 m 2 months, 9 m 9 months.

A proteomic analysis was conducted to figure out the roles of the unfolded protein response and ER stress in the pathogenesis of retinitis pigmentosa in *rd9* retinas. Figure 7A shows a diagram outlining the experimental strategy. Four groups were created according to the genotype and age of mouse retinas. Figure 7B shows that 53 proteins exhibited significant changes (*p* < 0.05 and FC > 1.5) at 2 months in *rd9* mouse retinas. Furthermore, 114 DEPs (*p* < 0.05 and FC > 1.5) were observed at 9 months in *rd9*. The Venn diagram indicated 4 overlapped DEPs in retinas at 2 m (yellow) and 9 m (green). In addition, there were 49 exclusive DEPs in 2 m retinas while 110 exclusive DEPs in 9 m retinas, illustrating the substantial alterations in 9 m *rd9* mouse retinas and the occurrence of retinal degeneration. This is consistent with the phenotype observed in *rd9* retinas at 9 m.

Principal component analysis (PCA) was conducted using normalized log2 intensity values for each protein to illustrate the overall protein differences between samples. Overall, the samples were divided based on genotype, WT versus *rd9* (PC1, 34.8% variance), and age, 2 months versus 9 months (PC2, 10.5% variance) (Fig. 7C). Additionally, clusters of *rd9*-2m and WT-2m overlapped partially, whereas *rd9*-9m and WT-9m clusters were well separated (Fig. 7D, E), highlighting WT and *rd9* retinas had notable differences at 9 m. Subsequently, to clarify the results, we present heatmaps of DEPs at 9 m in Fig. 7F. Red (high intensity) and blue (low intensity), which denote particular protein abundance, appeared to be clearly divided in the samples at 9 m. The columns are organized by genotype, demonstrating a clear segregation between *rd9* and WT samples at 9 months. The volcano plot showed 76 proteins were up-regulated (red) and 38 were downregulated (green) in *rd9* retinas at 9 months, with conditions of *p* < 0.05 and FC > 1.5 (Fig. 7G), where Cryaa was notably reduced.

DEPs (*p* < 0.05 and FC > 1.5) were enriched with DAVID for GO pathway analysis. As a result of GO-BP analysis, visual function pathways, also top 3 enriched pathways including lens development in camera-type eye, visual perception and sensory perception of light stimulus, were influenced severely at 9 m in *rd9* mice (Fig. 7H). The protein folding pathway was significantly affected at 9 months in *rd9* retinas, as evidenced by the corresponding heatmaps of DEPs, with Cryaa playing a substantial role. Likewise, the GO-MF analysis indicated a significant influence on the unfolded protein binding pathway, illustrating the important contribution of Cryaa. Furthermore, in the proteomic data of WT and *rd9* retinas at 9 m, unfolded protein response was enriched as the top entry for GSEA analysis, further indicating the critical role of ER stress in the pathogenesis of XLRP (Fig. 7I).

## Discussion

XLRP represents one of the most prevalent and severe genetic disorders that result in blindness, with the majority of cases attributed to mutations in the *RPGR* gene [28]. Despite its prevalence, the concrete pathological mechanisms driving retinal degeneration in XLRP remain incompletely defined. Understanding the molecular distinctions between degenerative and healthy retinas is crucial for elucidating the processes of retinal degeneration and may facilitate the development of novel therapeutic interventions.

Notably, the potential contribution of ER stress to XLRP pathogenesis has been largely unexplored. Few research has specifically focused on the key protein Cryaa in XLRP, particularly its function as a chaperone in preventing protein aggregation under diverse cellular stress conditions [29]. Specific mutations in proteins, such as rhodopsin, have been shown to result in abnormal protein folding and increased susceptibility to ER stress, thereby exacerbating retinal degeneration [30]. Additionally, progressive neurodegeneration in the *Pde6b* mutant *rd10* mouse model is associated with ER stress-mediated autophagy [31]. However, to the best of our knowledge, ER stress has not been studied in *Rpgr* mutant mouse models, and its contribution to retinal degeneration in this specific context remains unreported. Given that distinct genetic mutations drive diverse phenotypes and pathologic pathways, it is imperative to investigate ER stress in *rd9*, a *Rpgr* mutant mouse model, to elucidate the molecular mechanisms specific to XLRP.

In this study, we investigated the structural and functional disorders of the retina in *rd9* mice and identified the levels of ER stress-related proteins in both *rd9* mouse retinas and MEF-*rd9* cells. The up-regulation of CHOP (Ddit3) and GRP78 (BiP) confirmed the activation of UPR, indicating the early stages of ER stress. ER stress occurs when misfolded or unfolded proteins accumulate in ER lumen, triggering a series of cellular responses. In retinitis pigmentosa, multiple mechanisms may contribute to ER stress. While misfolded RPGR protein may initially trigger ER stress, our study highlights that downregulation of Cryaa serves to amplify this condition. In vitro knockdown of Cryaa in 661W cells confirmed its direct role in inducing ER stress. In the *rd9* model, Cryaa reduction appears to be a consequential event within the stress cascade initiated by the RPGR mutation, and its deficiency further aggravates imbalance in protein homeostasis, thereby driving apoptotic signaling. We therefore hypothesize that Cryaa acts as a critical mediator amplifying ER stress downstream of RPGR dysfunction. The exact mechanism underlying Cryaa downregulation remains to be determined and will be a focus of future investigation.

Our study represents the first examination at the organelle level in *rd9* mouse retinas and MEF-*rd9* cells. TEM revealed abnormalities in the ER and mitochondria, with the swollen ER confirming the presence of ER stress. The rapid swelling of the ER may be associated with an increased demand for membrane and ribosomes, potentially leading to the observed features [32, 33]. The mitochondrial alterations observed may result from interactions and exchanges of substances (eg. calcium ions) between the ER and mitochondria [34–36]. Furthermore, as a molecular chaperone reported to interact with 127 client proteins [37], Cryaa reduction may therefore destabilize the protein networks that facilitate ER-mitochondria communication. This could occur through the dysregulation of its client proteins involved in protein transport, organelle integrity, or stress signaling, thereby potentially contributing to disrupted proteostasis and cellular stress at the organelle interface.

In this research, MEF-*rd9* cells were chosen based on several key considerations. First, MEF-*rd9* cells are derived from *rd9* mice and possess the identical pathogenic mutation, thereby ensuring genetic consistency. Secondly, although photoreceptors are the primary affected cells in XLRP, obtaining pure primary photoreceptor cultures remains challenging due to their poor survival and proliferative capacity in vitro, as well as the high cellular heterogeneity of retinal tissue. In contrast, MEF cells are easy to obtain, culture and proliferate, making them a practical cell model. This study focuses on the fundamental pathological pathway of ER stress and apoptosis induced by Cryaa deficiency. We observed in MEF-*rd9* cells that: 1) decreased Cryaa protein expression; 2) marked ER swelling and ultrastructural alterations under TEM; and 3) changes in protein levels associated with ER stress and apoptosis. These findings confirmed MEF-*rd9* cells as a valid model. In addition, the use of fibroblasts to model neurodegenerative disease pathology is well-established and has led to key breakthroughs [38–40]. At present stage, using MEF-*rd9* cells for mechanism exploration is a necessary, efficient and rational strategy.

Furthermore, we conducted a proteomic analysis on age-matched WT and *rd9* retinas utilizing LC-MS/MS. This analysis revealed significant proteomic alterations in *rd9* retinas at 9 months of age. Despite visual perception and light stimulus pathways, protein folding and unfolded protein binding were the most affected pathways, where Cryaa was identified as a key factor. Notably, Hsp90, another protein known to be involved in the UPR [41, 42], was also implicated in these pathways. Additionally, functional enrichment analysis identified several pathways potentially associated with ER stress, including cytoplasmic microtubule organization, ATPase complex, Ran GTPase binding, nucleocytoplasmic carrier activity, and ATP-dependent protein binding.

ER is a critical organelle for maintaining protein homeostasis, a process especially vital in photoreceptors due to their high metabolic demand. In the *rd9* model, our data demonstrated that ER stress led to apoptosis, mediated in part by the up-regulation of CHOP. While previous studies reported that ER stress can also trigger autophagy and disrupt mitochondrial morphology and function [43–45], these specific pathways remain to be confirmed in *rd9* model. Furthermore, ER stress is known to interact with broader cell death and inflammatory pathways, such as ferroptosis and PAN-optosis. These processes represent plausible contributors to photoreceptor apoptosis in XLRP, and they may constitute important hypotheses for future investigation.

In conclusion, this study demonstrates that ER stress is a key driver of photoreceptor apoptosis in the *rd9* mouse model of XLRP and identifies Cryaa as a critical regulator within this pathway. These findings provide novel mechanistic insights into the pathogenesis of XLRP. The integrated proteomic and experimental data generated in this study establish a foundation for future therapeutic strategies aimed at modulating ER stress and protein homeostasis in retinal degeneration.

## Supplementary information

Figure S1

Figure S2

Table S1 siRNA sequences

Table S2 Quantitative proteomic profiling

Supplementary Figure and Table Legends

Original Western Blots

reproducibility checklist

## Data Availability

This article and supplementary information include all data supporting the findings of this study. The details of this study are available on request from the corresponding author. The mass spectrometry proteomics data have been deposited to the ProteomeXchange Consortium (http://proteomecentral.proteomexchange.org) via the iProX partner repository with the dataset identifier IPX0014981001.
