# Supplementary material for: Activation of ER stress mediated by HspB4/Cryaa reduction contributes to photoreceptor apoptosis in rd9 mouse model of X-linked Retinitis Pigmentosa
Source: Cell Death Dis. 2026 Aug 18;17(1):965. doi: 10.1038/s41419-026-09191-1 (PMC13759464; doi:10.1038/s41419-026-09191-1)
Supplement: Supplementary file 1 [file 41419_2026_9191_MOESM6_ESM.pdf]

Figure 3

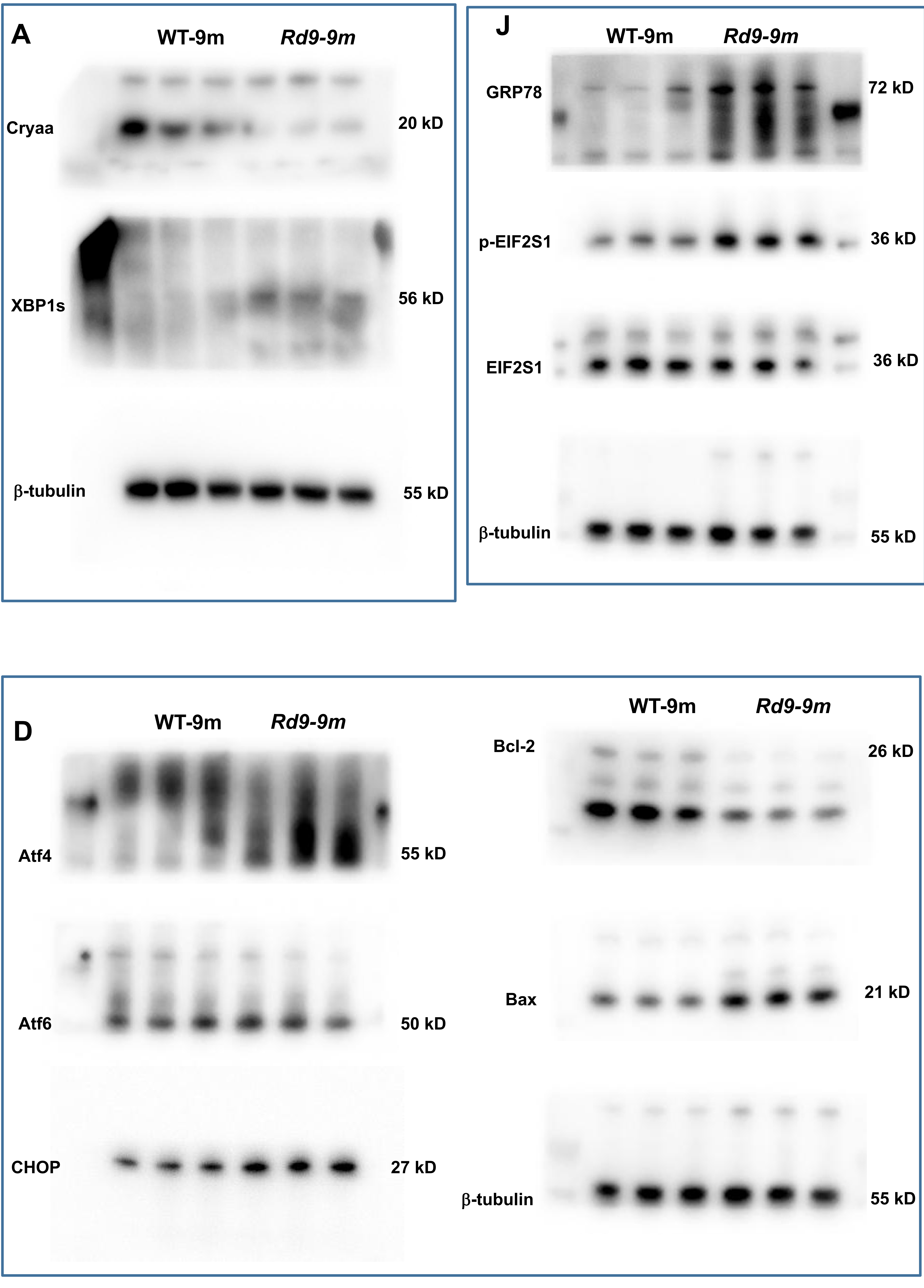
A
WT-9m
Rd9-9m
Cryaa
20 kD
XBP1s
56 kD
β-tubulin
55 kD
J
WT-9m
Rd9-9m
GRP78
72 kD
p-EIF2S1
36 kD
EIF2S1
36 kD
β-tubulin
55 kD
D
WT-9m
Rd9-9m
Atf4
55 kD
Atf6
50 kD
CHOP
27 kD
WT-9m
Rd9-9m
Bcl-2
26 kD
Bax
21 kD
β-tubulin
55 kD

Figure 5

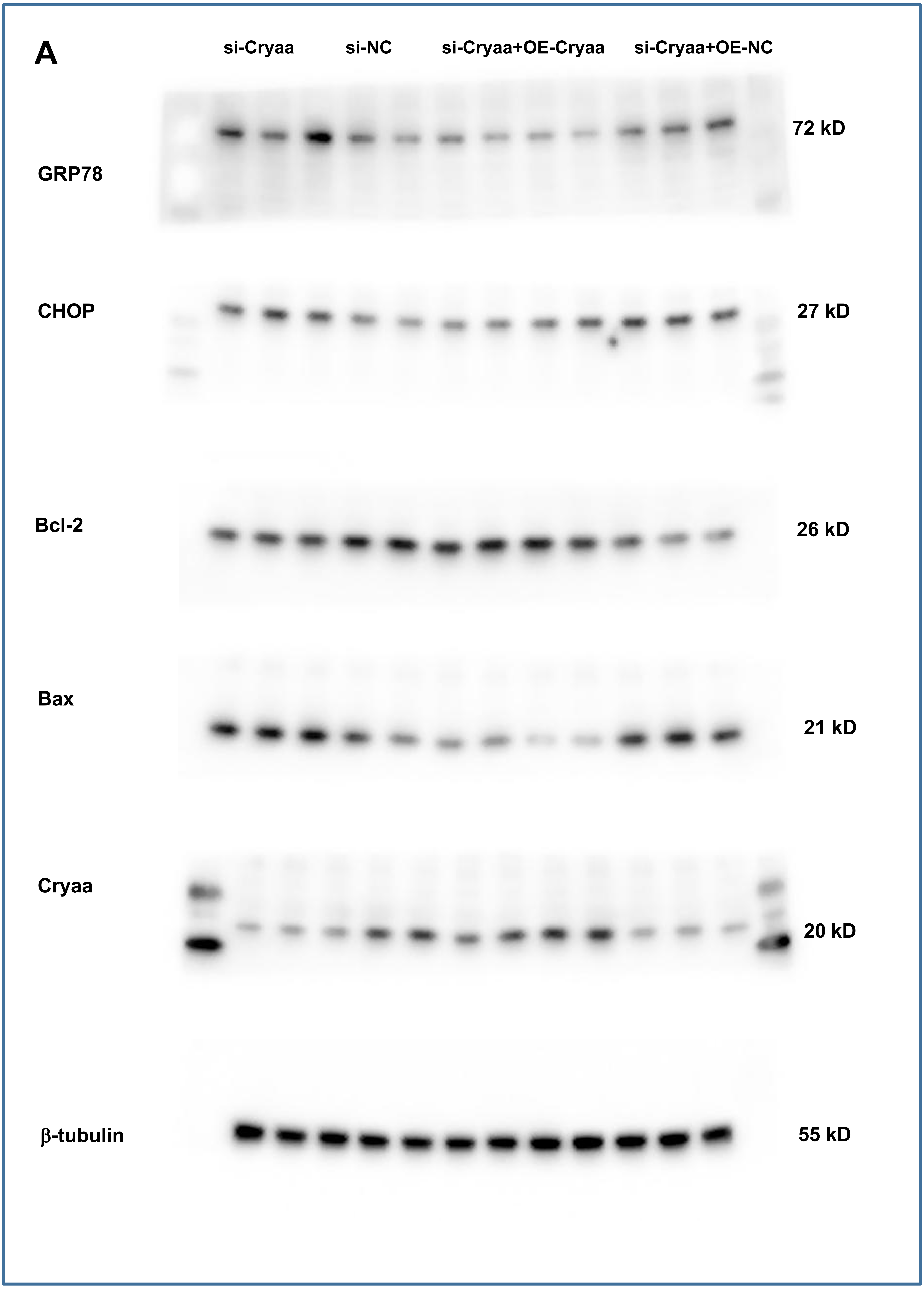
A
si-Cryaa
si-NC
si-Cryaa+OE-Cryaa
si-Cryaa+OE-NC
GRP78
72 kD
CHOP
27 kD
Bcl-2
26 kD
Bax
21 kD
Cryaa
20 kD
β-tubulin
55 kD

Figure 6

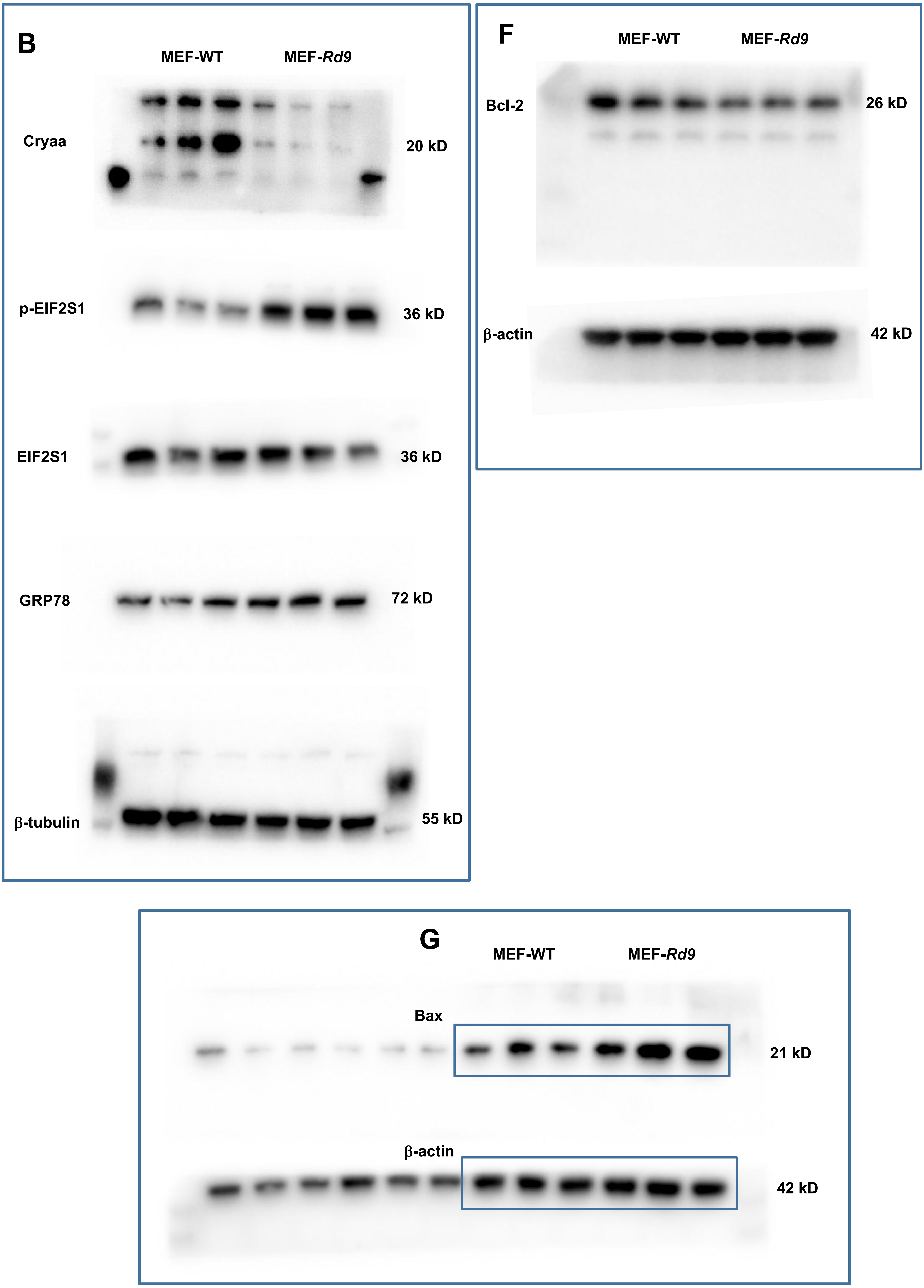
B
MEF-WT
MEF-Rd9
Cryaa
20 kD
p-EIF2S1
36 kD
EIF2S1
36 kD
GRP78
72 kD
β-tubulin
55 kD
F
MEF-WT
MEF-Rd9
Bcl-2
26 kD
β-actin
42 kD
G
MEF-WT
MEF-Rd9
Bax
21 kD
β-actin
42 kD

Figure 6

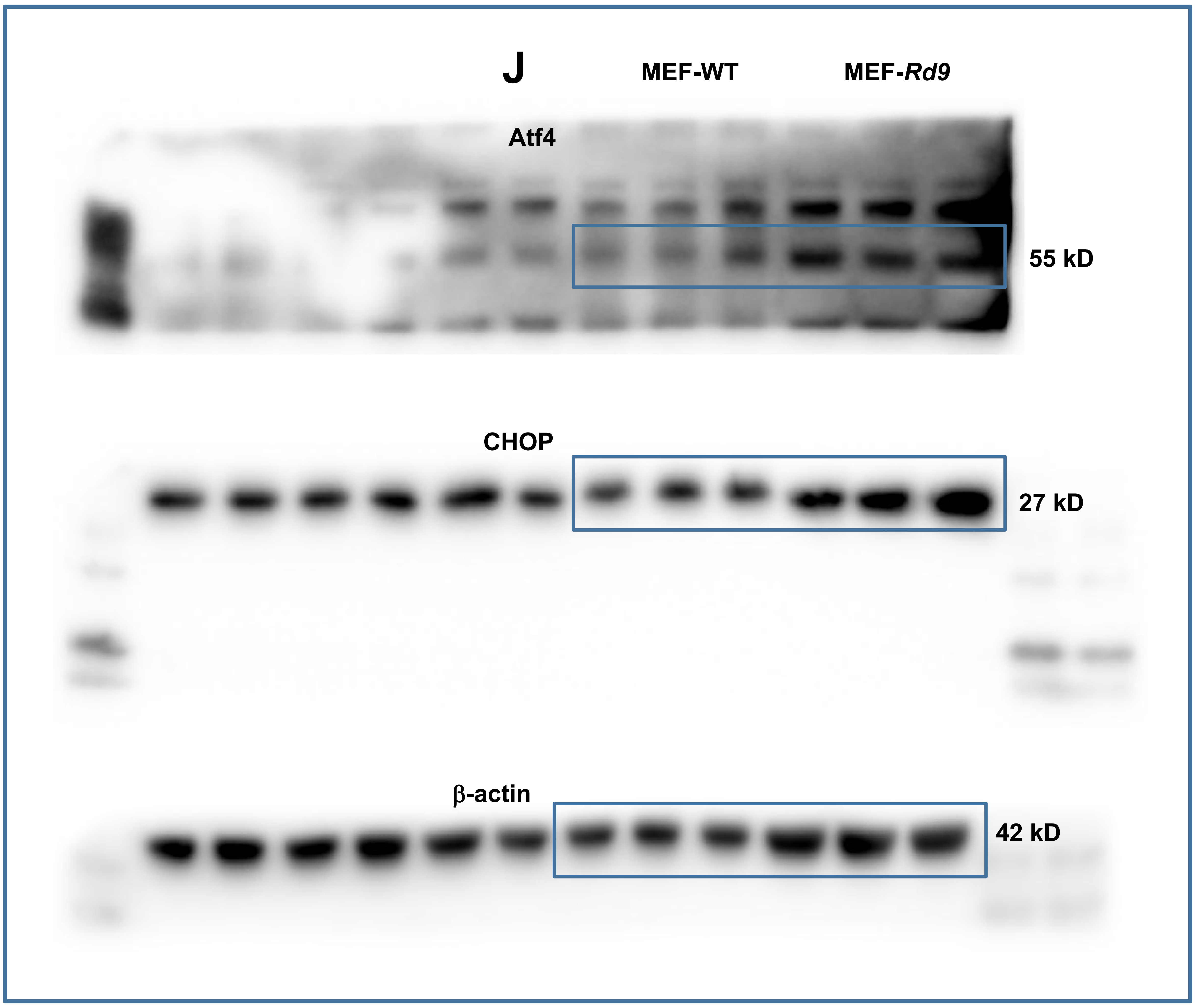

Figure S1

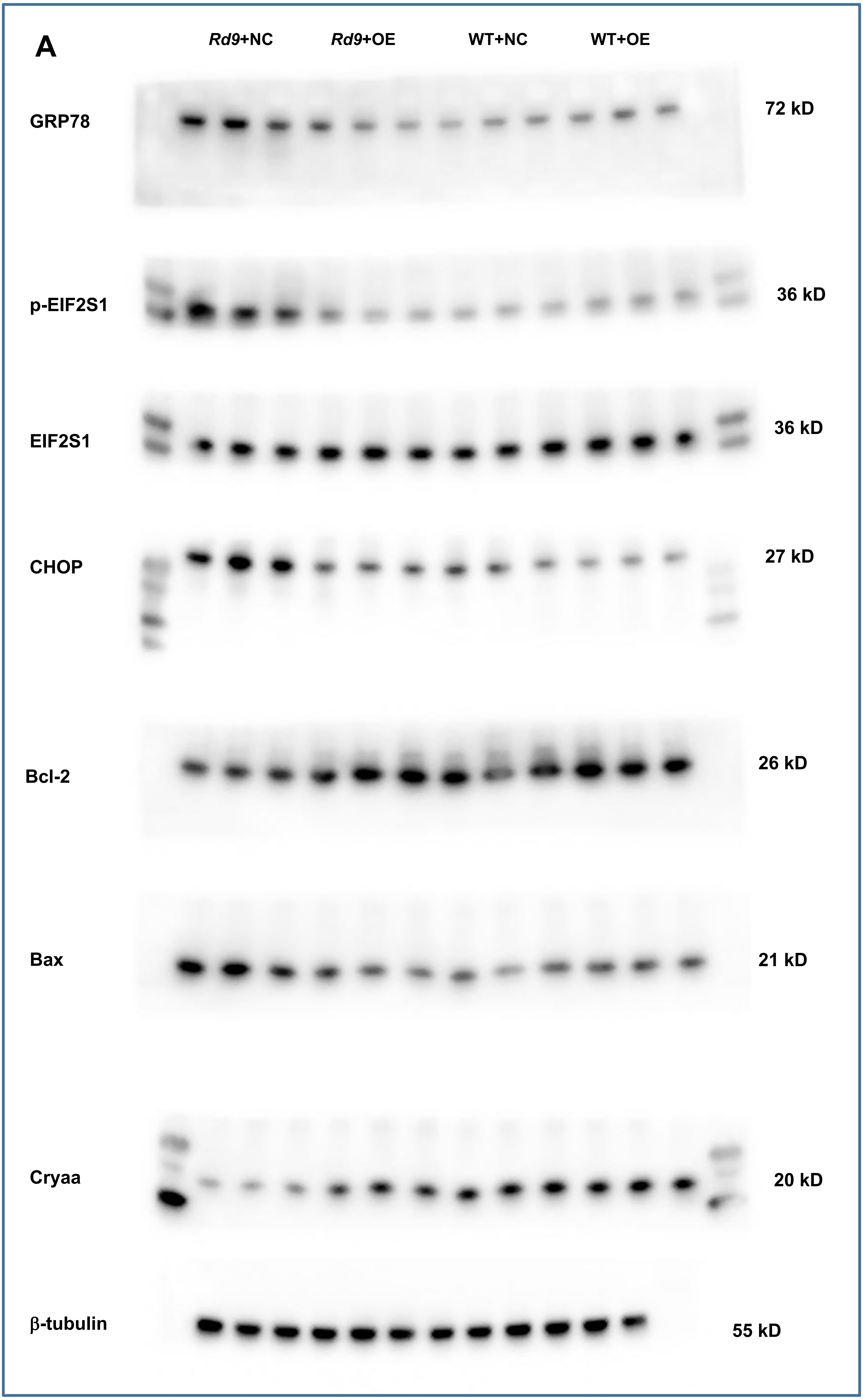
A
Rd9+NC
Rd9+OE
WT+NC
WT+OE
GRP78
72 kD
p-EIF2S1
36 kD
EIF2S1
36 kD
CHOP
27 kD
Bcl-2
26 kD
Bax
21 kD
Cryaa
20 kD
β-tubulin
55 kD
