# Supplementary material for: Activation of ER stress mediated by HspB4/Cryaa reduction contributes to photoreceptor apoptosis in rd9 mouse model of X-linked Retinitis Pigmentosa
Source: Cell Death Dis. 2026 Aug 18;17(1):965. doi: 10.1038/s41419-026-09191-1 (PMC13759464; doi:10.1038/s41419-026-09191-1)
Supplement: Supplementary file 2 [file 41419_2026_9191_MOESM7_ESM.pdf]

Corresponding Author Name: Xiuxiu Jin
Manuscript Number: JDIS-25-5615RR

SPRINGER NATURE

Reporting Summary

Springer Nature wishes to improve the reproducibility of the work that we publish. This checklist is used to ensure good reporting standards and to improve the reproducibility. Please respond completely to all questions relevant to your manuscript. For more information, please read the journal's Guide to Authors.

☑ **Check here to confirm that the following information is available in the Material & Methods section:**

- **The exact sample size (*n*)** for each experimental group/condition, given as a number, not a range
- **A description of the sample collection** allowing the reader to understand whether the samples represent technical or biological replicates (including how many animals, litters, culture, etc.)
- **A statement of how many times the experiment shown was replicated in the laboratory**
- **Definitions of statistical methods and measures**: For small sample sizes (n<5) descriptive statistics are not appropriate, instead plot individual data points
  - Very common tests, such as *t*-test, simple χ^2 tests, Wilcoxon and Mann-Whitney tests, can be unambiguously identified by name only, but more complex techniques should be described in the methods section
  - Are tests one-sided or two-sided?
  - Are there adjustments for multiple comparisons?
  - **Statistical test results**, e.g., ***P* values**
  - Definition of **'center values'** as **median or mean**;
  - Definition of **error bars as s.d. or s.e.m. or c.i.**

Please ensure that the answers to the following questions are reported in the manuscript itself. We encourage you to include a specific subsection in the methods section for statistics, reagents and animal models. Below, provide the page number or section and paragraph number.

Statistics and general methods

	**Reported in section/paragraph or page #**
1. How was the sample size chosen to ensure adequate power to detect a pre-specified effect size? (Give section/paragraph or page #)	Pages 5-6, lines 111-115
For animal studies, include a statement about sample size estimate even if no statistical methods were used.	Pages 5-6, lines 111-115
2. Describe inclusion/exclusion criteria if samples or animals were excluded from the analysis. Were the criteria pre-established? (Give section/paragraph or page #)	Page 6, lines 115-118
3. If a method of randomization was used to determine how samples/animals were allocated to experimental groups and processed, describe it. (Give section/paragraph or page #)	Page 6, lines 118-121
For animal studies, include a statement about randomization even if no randomization was used.	Page 6, lines 118-121

4. If the investigator was blinded to the group allocation during the experiment and/or when assessing the outcome, state the extent of blinding. (Give section/paragraph or page #)	Page 6, lines 121-124
For animal studies, include a statement about blinding even if no blinding was done.	Page 6, lines 121-124
5. For every figure, are statistical tests justified as appropriate?	Page 11-12, lines 242-245; Pages 29-32, lines 706-793
Do the data meet the assumptions of the tests (e.g., normal distribution)?	Page 11-12, lines 242-245; Pages 29-32, lines 706-793
Is there an estimate of variation within each group of data?	Page 11-12, lines 242-245; Pages 29-32, lines 706-793
Is the variance similar between the groups that are being statistically compared? (Give section/paragraph or page #)	Page 11-12, lines 242-245; Pages 29-32, lines 706-793

Reagents	**Reported in section/paragraph or page #**
6. Report the source of antibodies (vendor and catalog number)	Page 9, lines 182-186; Page 10-11, lines 219-222
7. Identify the source of cell lines and report if they were recently authenticated (e.g., by STR profiling) and tested for mycoplasma contamination	Page 7, lines 141-143

Animal Models	**Reported in section/paragraph or page #**
8. Report species, strain, sex and age of animals	Page 5, lines 103-107
9. For experiments involving live vertebrates, include a statement of compliance with ethical regulations and identify the committee(s) approving the experiments.	Page 5, lines 108-111

10. We recommend consulting the ARRIVE guidelines (*PLoS Biol.* **8**(6), e1000412,2010) to ensure that other relevant aspects of animal studies are adequately reported.

Human subjects

Reported in section/paragraph or page #

11. Identify the committee(s) approving the study protocol.	uninvolved
12. Include a statement confirming that informed consent was obtained from all subjects.	uninvolved
13. For publication of patient photos, include a statement confirming that consent to publish was obtained.	uninvolved
14. Report the clinical trial registration number (at ClinicalTrials.gov or equivalent).	uninvolved

15. For phase II and III randomized controlled trials, please refer to the CONSORT statement and submit the CONSORT checklist with your submission.

16. For tumor marker prognostic studies, we recommend that you follow the REMARK reporting guidelines.

Data deposition

Reported in section/paragraph or page #

17. Provide accession codes for deposited data. Data deposition in a public repository is mandatory for:<br>a. Protein, DNA and RNA sequences<br>b. Macromolecular structures<br>c. Crystallographic data for small molecules<br>d. Microarray data	Page 23, lines 504-506

Deposition is strongly recommended for many other datasets for which structured public repositories exist; more details on our data policy are available in the Guide to Authors. We encourage the provision of other source data in supplementary information or in unstructured repositories such as Figshare and Dryad. We encourage publication of Data Descriptors (see Scientific Data) to maximize data reuse.

18. If computer code was used to generate results that are central to the paper's conclusions, include a statement in the Methods section under "**Code availability**" to indicate whether and how the code can be accessed. Include version information as necessary and any restrictions on availability.	uninvolved
