# Supplementary material for: Activation of ER stress mediated by HspB4/Cryaa reduction contributes to photoreceptor apoptosis in rd9 mouse model of X-linked Retinitis Pigmentosa
Source: Cell Death Dis. 2026 Aug 18;17(1):965. doi: 10.1038/s41419-026-09191-1 (PMC13759464; doi:10.1038/s41419-026-09191-1)
Supplement: Supplementary file 3 [file 41419_2026_9191_MOESM3_ESM.docx]

|  | **Sense** | **Antisense** |
| --- | --- | --- |
| sc-40431A | UUCCUGUCUUCCACCAUCAtt | UGAUGGUGGAAGACAGGAAtt |
| sc-40431B | CUGAGCUCAUGACCCAUAUtt | AUAUGGGUCAUGAGCUCAGtt |
| sc-40431C | UGAAGGUACUGGAGGAUUUtt | AAAUCCUCCAGUACCUUCAtt |
| NC | UUCUCCGAACGUGUCACGUdTdT | ACGUGACACGUUCGGAGAAdTdT |

Note: all sequences are provided in 5’→3’orientation.
