# Supplementary material for: Activation of ER stress mediated by HspB4/Cryaa reduction contributes to photoreceptor apoptosis in rd9 mouse model of X-linked Retinitis Pigmentosa
Source: Cell Death Dis. 2026 Aug 18;17(1):965. doi: 10.1038/s41419-026-09191-1 (PMC13759464; doi:10.1038/s41419-026-09191-1)
Supplement: Supplementary file 4 [file 41419_2026_9191_MOESM5_ESM.docx]

**Supplementary Data**

Figure S1: Cryaa overexpression in MEF-*rd9* cells with MEF-WT as control. (A) (B) (C) Protein levels of GRP78, p-EIF2S1 and EIF2S1 in MEF-*rd9* cells transfected with pcDNA3.1(＋)/Cryaa. (D) (E) WB analysis of CHOP expression and ratio of Bax/Bcl-2 in MEF-*rd9* cells with pcDNA3.1(＋)/Cryaa transfection. (F) The expression levels of Cryaa in various groups. (n=3) The results were shown as mean±SEM. Significance of difference (**p*<0.05, **p<0.01) was determined by using one-way ANOVA with Bonferroni correction. NC: negative control with empty plasmid; OE: overexpression.

Figure S2: Schematic illustration shows the ER stress and apoptosis induced by misfolding Rpgr proteins and down-regulation of Cryaa in *rd9* mouse retina.

Table S1 Cryaa siRNA and NC siRNA sequences.

Table S2 Quantitative proteomic profiling of mouse retinas.
